# Drug conjugates for targeting regulatory T cells in the tumor microenvironment: guided missiles for cancer treatment

**DOI:** 10.1038/s12276-023-01080-3

**Published:** 2023-09-01

**Authors:** Juwon Yang, Hyunsu Bae

**Affiliations:** Department of Korean Medicine, College of Korean Medicine, Kyung Hee University, Seoul, Republic of Korea

**Keywords:** Cancer therapy, Tumour immunology

## Abstract

Within the tumor microenvironment (TME), regulatory T cells (Tregs) play a key role in suppressing anticancer immune responses; therefore, various strategies targeting Tregs are becoming important for tumor therapy. To prevent the side effects of nonspecific Treg depletion, such as immunotherapy-related adverse events (irAEs), therapeutic strategies that specifically target Tregs in the TME are being investigated. Tumor-targeting drug conjugates are efficient drugs in which a cytotoxic payload is assembled into a carrier that binds Tregs via a linker. By allowing the drug to act selectively on target cells, this approach has the advantage of increasing the therapeutic effect and minimizing the side effects of immunotherapy. Antibody–drug conjugates, immunotoxins, peptide–drug conjugates, and small interfering RNA conjugates are being developed as Treg-targeting drug conjugates. In this review, we discuss key themes and recent advances in drug conjugates targeting Tregs in the TME, as well as future design strategies for successful use of drug conjugates for Treg targeting in immunotherapy.

## Cancer immunotherapy: how to selectively eliminate immune-suppressive regulatory T cells

Targeted drug delivery systems offer a promising strategy for specifically depleting immune cells in the tumor environment known as regulatory T cells (T_reg_) that dampen the body’s anti-cancer responses and promote tumor growth. Juwon Yang and Hyunsu Bae from Kyung Hee University in Seoul, South Korea, review how potent cytotoxic drugs linked to targeting molecules can help to eliminate T_reg_ cells in the tumor vicinity without the off-target effects that can cause systemic side effects. Diverse types of drug designs are being used to target these immune-suppressive cells, including antibody–drug conjugates, immunotoxins, peptide–drug conjugates and small interfering RNA conjugates. Targeted elimination of T_reg_ cells with these technologies could help to enhance the function of other tumor-killing T cells and improve the overall efficiency of immunotherapies for cancer patients.

## Introduction

**Fig. 1:**
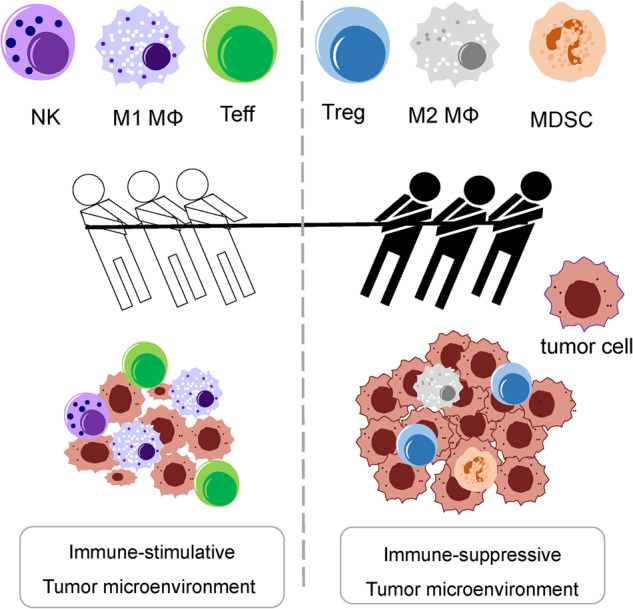
Illustrative explanation of mutual dynamics between the immunostimulatory and immunosuppressive tumor microenvironment (TME). Regulatory T cells (Tregs), M2 macrophages (MΦ), and myeloid-derived suppressor cells (MDSCs) are predominant in the immunosuppressive TME during tumor development.

Over the past few years, advancements in anticancer immunotherapies have increasingly aided treatment of solid and hematological malignancies. Immune checkpoint inhibition (ICI) and adoptive transplantation of genetically engineered T cells (CAR-T cells) have been successfully used to treat malignancies. However, there are some obstacles to the current strategy of immunotherapy: (1) lack of T-cell access due to disorganized neovasculature and stromal barriers, tumor antigens and the mutational burden, and tumor heterogeneity and (2) T-cell exhaustion due to suppressive tumor-associated macrophages (TAMs), regulatory T cells (Tregs), and hypoxia in the suppressive tumor microenvironment (TME) (Fig. 1). Therefore, new approaches have been adopted to overcome these challenges. For example, TAMs, Tregs, immunosuppressive cytokines, and hypoxia have been targeted to enhance T-cell access and overcome TME-mediated immune suppression^1^.

The TME is similar to the play Othello; it is similar to a zero-sum game. On a game board, antitumor immune cells and immunosuppressive actions strongly oppose each other. White discs, such as effector T cells, M1 macrophages, and natural killer cells, compete with black discs, such as Tregs, M2 macrophages, and tumor cells. All the cells play strategically. When an effector T-cell kills a tumor cell, similar to turning a black disc into a white disc, the Tregs hidden in the diagonal corners turn the white disc all at once and cause numerous effector T cells to disappear instantly (Fig. 1).

Tregs accumulate at high rates in the immunosuppressive TME during tumor development. They activate effector T-cell suppression by the following mechanisms: (1) regulation of antigen-presenting cell (APC) function through competitive blockade of CD80 and CD28 binding costimulation by CTLA-4, (2) secretion of immunosuppressive molecules such as IL-10 and TGF-β, (3) suppression of effector T-cell growth by inducing depletion of the local IL-2 pool due to high CD25 (IL-2Ra) expression, and (4) secretion of perforins and granzymes to cause apoptosis of effector T cells. Therefore, the effectiveness of immunotherapy can be enhanced by targeting Tregs, enabling antitumor treatment^1,2^.

A drug conjugate combines a cytotoxic drug with a carrier capable of binding to a specific target using chemical linkers. Drug conjugates are being actively developed for targeted delivery to specific regions and effectively kill target cells with remarkable potency, even at small dosages. Recently, drug conjugates targeting Tregs have been developed and shown to be successful^3^. They represent innovative immunotherapeutic agents and act by altering the immunosuppressive environment in a manner different from the existing method of targeting cancer cells^4^.

In this review, we focus on the immunosuppressive TME and discuss current approaches for targeting/depleting Tregs to overcome TME-mediated immune suppression. In addition, we review drug conjugates for targeting/depleting Tregs in novel trials.

## Immunosuppressive cells in TME

The TME is a highly complex and heterogeneous ecosystem that includes malignant cells and host-interacting cells such as endothelial cells, stromal fibroblasts, and various immune cells. In this ‘nest’, tumor cells undergo differentiation, epigenetic changes, dissemination, and immune evasion^5^.

Transformation of normal cells into tumor cells relies on irreversible genetic alterations that trigger oncogenic signaling pathways. According to Swann and Smyth, “Transformed cells that escape intrinsic control are subjected to extrinsic tumor suppressor mechanisms that detect and eliminate developing tumors before they become clinically apparent.” This is the elimination phase of a broader process known as cancer immunoediting^6^.

Immunoediting consists of immune surveillance and tumor progression in three stages: elimination, equilibration, and escape. The immune elimination phase includes both innate and adaptive immune responses against tumor cells. Most tumor cells are destroyed at this stage; however, some can survive and reach equilibrium with the immune system. During the equilibrium phase, tumor cells with a nonimmunogenic phenotype are selected and grow. Tumor cell variants that acquire clearance resistance enter the escape phase. During the escape phase, tumor cells continue to grow and expand in an uncontrolled manner, eventually leading to malignancy^7^.

Tumor immunogenicity is modified throughout these phases, and immunosuppressive mechanisms that enable disease progression are acquired^8^. The immunosuppressive TME is enriched in immunosuppressive cells such as Tregs, TAMs, myeloid-derived suppressor cells, tumor-associated neutrophils, and tumor-associated dendritic cells (Fig. 1). Cytokines and related molecules secreted by these cells and tumor cells promote tumor progression and mediate immune escape^9^.

## Tregs in the TME and specific markers

Tregs are one of the representative immunosuppressive cells and functionally ambivalent. They have several positive roles, including a role in immune tolerance, inhibition of autoimmune diseases, prevention of tissue damage, and regulation of inflammation after infection; negative roles include interference with cancer immunity. Tregs are chemoattracted to the TME by a chemokine gradient secreted by tumor cells and are then activated to suppress the antitumor immune response and release immunosuppressive cytokines such as IL-10, TGF-β, and IL-35. In addition, Tregs suppress other immune cells, such as basophils, eosinophils, and mast cells, and release perforins and granzymes upon binding to T-cell receptors that target cytolysis of effector T cells and APCs^5^.

The Sakaguchi group noted in a recent review paper that “Tregs are one of the important roadblocks in tumor treatment, so it is necessary to remove them before tumor defeat”^10^. Targeting Tregs is an efficient strategy for cancer treatment; however, nonspecific systemic Treg depletion causes problems such as immunotherapy-related side effects. Hence, there are several criteria for selecting Tregs in tumors through their specific markers for targeting.

Sakaguchi et al. classified CD4 + FOXP3 + T cells in humans into the following three subsets based on expression of CD45RA, a cell surface marker of naïve T cells, and the transcription factor FOXP3: fraction 1 (Fr. 1) naïve Tregs, as defined as FOXP3^low^ (CD25^low^) CD45RA^+^ cells; fraction 2 (Fr. 2) effector Tregs (eTregs), as defined as FOXP3^high^ (CD25^high^) CD45RA^−^ cells; and fraction 3 (Fr. 3), and non-Tregs, as defined as FOXP3^low^ (CD25^low^) CD45RA cells^11^.

Naive Tregs (Fr. 1) are Tregs that leave the thymus and have weak immunosuppressive activity. Upon receiving T-cell receptor stimulation, they differentiate into eTregs (Fr. 2), which have strong immunosuppressive activity. In most cancers, the frequency of eTregs is 2–5% in peripheral blood but 10–50% in tumor tissue; thus, they are mostly distributed in tumor tissue. Non-Tregs (Fr. 3) do not have immunosuppressive properties and produce inflammatory cytokines such as interferon (IFN)-γ and IL-17^12^.

**Fig. 2:**
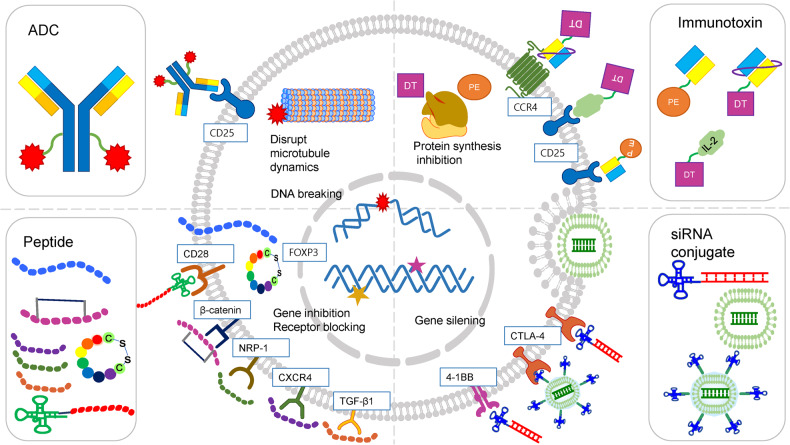
Illustration of the mechanism of action of drug conjugates targeting regulatory T cells (Tregs). ADC antibody–drug conjugate, DT diphtheria toxin, PE Pseudomonas exotoxin A, siRNA small-interfering RNA.

Tregs in the TME have several membrane targets, such as CD25, CTLA-4, PD-1, ICOS, GITR, OX40, CCR4, and CCR8, that can be exploited to deplete these cells. Treg-targeting drug conjugates target several of these markers (Fig. 2)^13^.

CD25 is an IL-2 receptor, and IL-2 is an important factor for T-cell maintenance. As eTregs are CD25^high^ cells, CD25 can be targeted to deplete eTregs. CD25 has been used as a target for the antibody–drug conjugate (ADC) ADCT-301 as well as the immunotoxins 2E4-PE38, Denileukin Diftitox, and LMB-2^3,14–16^.

CTLA-4 is an important molecule for Tregs and functions as an immune checkpoint protein that negatively regulates T-cell activation and contributes peripherally. Tregs express CTLA-4 to suppress APCs. CTLA-4 binds to CD80 and CD86 with higher affinity than CD28 and acts as a competitive inhibitor of CD28 on APCs. It has been used as a target for small interfering RNA (siRNA) conjugates (CTLA4apt–STAT3 siRNA, NPsiCTLA-4, cSNPs, and hybrid SNPs)^17–19^.

Because Tregs express costimulatory receptors such as GITR, OX40, and ICOS, targeting them can lead to Treg depletion and functional regulation. GITR, which plays an essential role in Treg expansion, is expressed at high levels by Tregs but at low levels by resting CD4+ and CD8 + T cells. OX40 is constitutively expressed by a subset of Tregs but is also found on effector T cells. ICOS, which is essential for Treg function and homeostasis, is highly expressed by activated Tregs among the tumor-infiltrating lymphocytes (TILs) of patients with gastric cancer^13^.

Chemokine receptors that allow Tregs to migrate to the TME are candidate molecules for Treg depletion. CCR4 is highly expressed by eTregs but not by most effector T cells, except for naive Tregs and some Th2 and Th17 cells in peripheral blood^13^. CCR8 is a chemokine receptor expressed at high levels on the surface of tumor-infiltrating Tregs but not on peripheral Tregs or effector T cells^20–22^. Reanalysis of T-cell scRNA-seq datasets of non-small cell lung cancer^23^ and colorectal cancer^24^ has shown that CCR4 and CCR8 are more selectively expressed in tissue-associated Tregs than in Tregs in peripheral or healthy tissues^25^.

FOXP3 is considered a master regulator of the immunosuppressive phenotype of Tregs^26^. Naive and memory CD4 + T cells differentiate into Tregs by inducing FOXP3 expression^27^. FOXP3 is a promising target for Treg suppression; however, because of its nuclear localization, targeting requires strategies or efficient delivery of suppressive molecules into cells^28^. Cell-penetrating peptides or short RNA strands can be used as efficient FOXP3 target molecules. P60, CM1315, and FOXP3 393–403 are peptides developed to target FOXP3^29,30^. siRNAs targeting FOXP3 are predesigned and marketed for use in gene silencing^31^, or they can be designed and produced directly based on target sequences^32^. Manrique-Rincón et al. synthesized a 4-1BB aptamer conjugated with a small antisense RNA (sasRNA) for *Foxp3* silencing in melanoma-bearing mice^28^.

## Antibody–drug conjugates

ADCs are drug conjugates in which monoclonal antibodies (mAbs) are coupled to cytotoxic payloads via a linker (Fig. 2). Since Paul Ehrlich coined the term “magic bullet” 100 years ago, postulating that some compounds can directly access a desired target in cells to treat diseases, ADC use has become an important option in cancer treatment^33^.

The ADC gemtuzumab ozogamicin (Mylotarg®) was approved for the first time by the US Food and Drug Administration (FDA) in 2000. To date, 14 ADCs have been approved, and more than 100 ADC candidates are currently in the clinical stage^34^.

Monoclonal antibodies are specific for cell surface targets, provide specificity and efficacy, and can be used to deplete or disrupt Treg function^35^. Treg-targeting mAbs such as daclizumab (CD25), ipilimumab (CTLA-4), nivolumab (Pd-1), and mogamlizumab (CCR4) are used to induce antitumor immunity, including antibody-dependent cytotoxicity, complement-dependent cytotoxicity, and antibody-dependent cellular phagocytosis effects^36^.

Linkers play a pivotal role in the stability and homogeneity of ADCs by maintaining their stability in systemic circulation and releasing the payload after internalization at the target site. Unstable linkers cause systemic toxicity by releasing small cytotoxic molecules into the bloodstream. Linkers are subdivided into two categories based on their mechanism of payload release: cleavable and noncleavable^37^.

Payloads are largely divided into DNA-damaging agents (e.g., pyrrolobenzodiazepines [PBDs], calicheamicins, duocarmycins, and SN-38) and microtubule-disrupting agents (e.g., auristatins and maytansines)^38^. Calicheamicins, duocarmycins, and PBDs are natural antibiotics that bind to DNA minor grooves and cause strand breaks^39^; SN-38 inhibits TOP1 (topoisomerase I), causing S-phase-specific cytotoxicity and mediating DNA single-strand breaks^40^. Auristatin monomethyl auristatin E (MMAE) and monomethyl auristatin F (MMAF) induce G2/M phase cell cycle arrest by interfering with microtubule polymerization when binding to the β-subunit of the tubulin dimer^41^., DM1 (a derivative of maytansine 1) is a maytansinoid used in T-DM1^42^.

A good ADC has a high internalization rate, low immunogenicity, high binding specificity and affinity, a strong payload, and a stable linker. It provides improved efficacy compared to traditional antibody drugs and enables targeted delivery of cytotoxic drugs. Although ADCs have been developed to limit toxicity, with progression of clinical trials, the limitations of ADCs, such as hepatic, neurological, and ocular toxicity as side effects, have been noted, as has drug resistance due to payload internalization and retention^37^.

## ADCs targeting Tregs in the TME

The existing ADCs were mainly developed to target cancer cells directly. However, the study of Zammarchi et al. relied on ADCs that directly target immune cells rather than tumor cells, providing a proof-of-concept for an entirely new application of ADCs as immunotherapeutics. Using camidanlumab tesirine (ADCT-301), which targets human CD25 based on a pyrrolobenzodiazepine (PBD) dimer, the authors demonstrated that Treg depletion and antitumor immunity can eradicate tumors (Fig. 2).

Camidanlumab tesirine (ADCT-301) is being evaluated in several clinical trials (Table 1). These studies include the following: a phase 2 clinical trial in patients with relapsed or refractory Hodgkin’s lymphoma (NCT04052997)^43^; a phase 1 with relapsed or refractory Hodgkin’s lymphoma and non-Hodgkin’s lymphoma (NCT02432235)^44^; a phase 1 in acute myeloid leukemia or acute lymphoblastic leukemia (NCT02588092)^45^; and a phase 1 in solid tumors (NCT03621982)^4^.

**Table 1:** Summary of drug conjugates targeting Tregs in the TME.

Class of drug conjugates	Target	Name	Homing molecules	Cytotoxic payload	Mechanism of action	Condition	Clinical/preclinical	Refs.
ADC	CD25	Camidanlumab tesirine (ADCT-301)	Anti-CD25 mAb	Pyrrolobenzodiazepine (PBD)	Binding in the minor groove of DNA and linking the two DNA strands together	Hodgkin/non-Hodgkin lymphoma	Phase 2 NCT04052997	^43^
						Hodgkin/non-Hodgkin lymphoma	Phase 1 NCT02432235	^44^
						Acute myeloid leukemia/Acute lymphoblastic leukemia	Phase1 NCT02588092	^45^
						Solid tumors	Phase 1 NCT03621982	^4^
Immunotoxin	CD25	Anti-Tac(Fv)‐PE38(LMB-2)	Fv of anti-Tac (CD25) mAb	Pseudomonas exotoxin A	Inhibition of protein synthesis	Adult T-cell lymphoma	Phase 2 NCT00924170	^55^
						Hairy cell Leukemia	Phase 2 NCT00321555	^56^
	CD25	Denileukin diftitox (DAB389-IL-2,Ontak®)	Interleukin-2	Diphtheria toxin	Inhibition of protein synthesis	Cutaneous T-Cell Lymphoma (CTCL)	FDA-approved	^57^
	CD25	Denileukin diptitox (s-DAB-IL-2 (V6A))	Interleukin-2	Diphtheria toxin	Inhibition of protein synthesis	Melanoma	Preclinical studies	^58^
	CD25	Denileukin diptitox (E7777(I/ontak))	Interleukin-2	Diphtheria toxin	Inhibition of protein synthesis	Persistent or Recurrent Cutaneous T-Cell Lymphoma	Phase 3 NCT01871727	
						Peripheral/Cutaneous T-cell Lymphoma	Phase 2 NCT02676778	^60^
	CD25	2E4-PE38	Fv of Rat anti- CD25 mAb (2E4)	Pseudomonas exotoxin A	Inhibition of protein synthesis	Mesothelioma, breast cancer, colon cancer	Preclinical studies	^3^
	CCR4	DT390‐BiscFv(1567)‐6xHis	scFv of anti-CCR4 mAb(mAb1567)	Diphtheria toxin (DT390)	Inhibition of protein synthesis	In vitro studies in human PBMC	Preclinical studies	^63^
	CCR4	bispecific human IL2‐CCR4	scFv of anti-CCR4 mAb,IL-2	Diphtheria toxin (DT390)	Inhibition of protein synthesis	Cutaneous T‐cell lymphoma	Preclinical studies	^64^
siRNA	CTLA-4, STAT	CTLA4apt–STAT3 siRNA	CTLA4 binding RNA aptamer	siRNA-targeting STAT3	Stat3 silencing	T-cell lymphoma	Preclinical studies	^17^
	CTLA-4	NPsiCTLA-4	Nanoparticles composed of PEG5k–PLA11k and BHEM-Chol	siRNA-targeting CTLA-4	CTLA-4 silencing	Melanoma	Preclinical studies	^18^
	CTLA-4, PD-1	pSNPs & cSNPs, hybrid SNPs	Nanoparticle composed of PLGA-S-S-PEG core and cationic lipid DOTAP	Aptamer-targeting CTLA-4,siRNA-targeting PD-1,Both	CTLA-4 silencing, PD-1 silencing, Both	Melanoma, Colorectal adenocarcinoma	Preclinical studies	^19^
sasRNA	4-1BB, FOXP3	4-1BB-TS6 aptamer	4-1BB binding aptamer	small antisense RNA (sasRNA) targeting FOXP3	FOXP3 silencing	Melanoma	Preclinical studies	^28^
Peptide	FOXP3	AptCD28-P60	CD28 binding aptamer	P60	Preventing foxp3 nuclear translocation	Colon cancer	Preclinical studies	^75^

## Immunotoxins

An immunotoxin is an immunoconjugate that induces death of a target cell by combining an antibody with target-specific high-affinity binding activity with other molecules, such as radioisotopes, chemicals, siRNA, and cytotoxic proteins (Fig. 2)^46^. To date, three cytotoxins and immunotoxins, namely, Denileukin diftitox (Ontak®)^15^, Tagraxofusp (Elzonris®)^47^, and Moxetumomab pasudotox (Lumoxiti®)^48^, have been FDA-approved for treatment of several forms of hematological cancer, and more than 20 treatments are being clinically tested^49^.

Immunotoxins have a mechanism of action similar to that of ADCs, in which fragments of immunotoxin mAbs bind to the target cell surface, and the protein toxin enters the cell and interferes with cellular processes; however, in contrast to ADCs, immunotoxins have potent protein toxins as payloads and can effectively kill quiescent, nondividing cells^50^. The payloads used for immunotoxins are cytotoxic proteins derived from bacteria, plants, or humans. Representative examples include *Pseudomonas aeruginosa* exotoxin A (PE), diphtheria toxin (DT), ricin, saporin, gelonin, proteases, and ribonucleases (RNases)^46^. ADCs can induce off-target toxicity due to improper payload separation from chemical linkers; however, modern recombinant immunotoxins do not have such problems because specific intracellular proteases to separate the recombinant peptide linkers are required^51^.

Immunotoxins can induce vascular leak syndrome through enzymatic activity against the endothelium. As they are immunogenic molecules, anti-immunotoxin antibodies are produced when administered to patients. Various strategies have been studied to overcome this problem^51^. Because immunotoxins are relatively large molecules, they cannot readily penetrate solid tumors. For improved solid tumor penetration and antitumor efficacy, the target moiety of an immunotoxin is usually a single-chain variable fragment (scFv) or a novel scaffold^52^.

## Immunotoxins for targeting Tregs in the ***TME***

Anti-Tac (Fv)‐PE38 (LMB-2) is a recombinant immunotoxin composed of a variable domain (Fv) of antibody binding to Tac (CD25) and *Pseudomonas* exotoxin A (PE38). LMB-2 is an effective treatment for hairy cell leukemia^53^. LMB-2 can eliminate Tregs expressing CD4, CD25, and FOXP3 in humans but not in mice^54^. Powell et al. studied the improvement in the clinical effect of immunotherapy in patients with melanoma by inducing elimination of Tregs by administering LMB-2 and demonstrated the capacity of a CD25-directed immunotoxin to selectively mediate a transient partial reduction in circulating and tumor-infiltrating Tregs in vivo^16^. A phase 2 trial of LMB-2—Fludarabine and Cyclophosphamide for Adult T-Cell Leukemia— (NCT00924170)^55^ and a phase 2 trial —LMB-2 to Treat Hairy cell Leukemia— (NCT00321555)^56^ are ongoing (Table 1).

Denileukin diftitox (DAB389-IL-2, Ontak®) is an engineered immunotoxin combining IL-2 and diphtheria toxin^46^. It was FDA-approved in 2008 for treatment of persistent or recurrent cutaneous T-cell lymphoma and has been used to eliminate CD25+ lymphoma cells and Tregs^57^. However, its development was stopped in 2011 due to severe toxicity, and it was voluntarily withdrawn from the market in 2014 due to manufacturing difficulties. Second-generation denileukin diptitox (s-DAB-IL-2 (V6A)) with reduced vascular leakage was developed by Cheung et al. ^58^, and E7777 (I/Ontak), a purified version of denileukin diptitox, was developed. E7777 is undergoing clinical research^59^. The phase 3 trial “Persistent or Recurrent Cutaneous T-Cell Lymphoma” (NCT01871727) and phase 2 trial “Peripheral/Cutaneous T-cell Lymphoma” (NCT02676778) have been completed^60^. “T-regulatory Cell Depletion With E7777 Combined With Pembrolizumab in Recurrent or Metastatic Solid Tumors” is a phase 2 trial in the recruiting stage (NCT05200559).

2E4-PE38 is a single-chain recombinant anti-CD25 immunotoxin in which the Fv portion of rat mAb 2E4 is genetically fused to a 38-kDa fragment of PE and has strong cytotoxic activity. Onda et al. treated mice with breast cancer, mesothelioma, and colon cancer by intratumoral injection of 2E4-PE38 to kill Tregs and found that the immunotoxin caused complete regression of the treated tumors, as well as most distal noninjected tumors, inducing systemic antitumor immunity^3^.

DT390‐BiscFv(1567)‐6xHis is a diphtheria toxin-based recombinant anti-human CCR4 immunotoxin. Wang et al. developed it using the scFv of an anti-CCR4 monoclonal antibody (mAb1567)^61^ and diphtheria toxin (DT390)^62^. They demonstrated the binding and in vivo efficacy of DT390‐BiscFv(1567)‐6xHis on nonhuman primate (NHP) CCR4^+^ FOXP3^+^ monkey Tregs. Its administration in monkeys led to depletion of 78-89% of CCR4^+^ FOXP3^+^ monkey Tregs for 10 days, while not affecting other cells, including CD8^+^ T cells, B cells, and natural killer cells^63^. Wang et al. subsequently synthesized an IL2‐CCR4/CCR4‐IL2 bispecific immunotoxin by recombining Ontak®‐like human IL2 fusion toxin and ccr4 immunotoxin. They demonstrated that the efficacy of bispecific human IL2‐CCR4 immunotoxin in mouse cutaneous T-cell lymphoma (CTCL) was more effective than when used alone^64^.

## Peptide–drug conjugates

Peptide–drug conjugates (PDCs) are next-generation targeted therapies that were developed after ADCs. Currently, there are two drugs approved for marketing by the FDA^65^.

Because most mAbs have a molecular weight of ~150 kDa, they are too large to infiltrate tumor tissues. They may be immunogenic and aggregate in excretory organs, such as the kidney and liver^66–68^. Additionally, mAb generation is expensive and time-consuming^69^.

Peptides well penetrate solid tissues due to their low molecular weight and are considered safe due to their low immunogenicity and nontoxic metabolite production^70^. They are also inexpensive, easily synthesized, and transformable^71^.

PDCs consist of a homing peptide, payload, and linker. The peptides in the PDC are target cell specific and should induce receptor-mediated endocytosis of the conjugate. Payloads include highly toxic maytansine, camptothecin derivatives, auristatin, or doxorubicin. Linkers are chosen to allow sufficient circulation time for the conjugate to reach the target cell^72^.

The peptides used in PDC can be broadly divided into two categories: cell-penetrating peptides (CPPs) and cell-targeting peptides (CTPs). CPPs use specific amino acid sequences to effectively transport cell-impermeable compounds or drugs across cell membranes to reach their intracellular targets. CTP is an ideal homing molecule because, similar to mAbs, it binds with high affinity to receptors overexpressed on the target cell surface. Most homing peptides are linear and bind well but have several drawbacks, including enzymatic degradation at the termini, chemical instability, and fast renal clearance. To compensate for this, cyclization or peptide stapling of linear peptides has been used, as have conjugation to gold nanoparticles and antibody Fc or albumin^73,74^.

Peptides can be selected by screening peptide libraries according to their binding affinity to the target. Peptide libraries are systematic combinations of diverse peptides widely used to identify gene- or cell-targeted therapies for cancer treatment. Depending on the type and method of displaying the target, such libraries include ribosome display, phage display, mRNA display, and protein fragment complementation analyses^30^.

## Peptides for targeting Tregs in the ***TME***

Until now, development of PDCs as a Treg target has not been reported, but studies on peptides that inhibit Tregs are being actively conducted. CPPs can be used as a payload in a drug conjugate, and P60 conjugated with a CD28 aptamer is a good example^75^. CTPs with high binding affinity to the Treg membrane target have the potential to be used as a homing molecule for PDCs. Studies on peptides for targeting Tregs are mostly in the preclinical stage (Table 2).

**Table 2:** Summary of peptides targeting Tregs in the TME.

Name	Target	Sequence	Mechanism of action	Condition	Refs.
P60	FOXP3	RDFQSFRKMWPFFAM	Preventing foxp3 nuclear translocation	Colon cancer	^76^
CM-1315-P60	FOXP3	Cyclic [RDAFQAFRKMWPFFAM]	Preventing foxp3 nuclear translocation	Colon cancer	^77^
FOXP3 393–403	FOXP3	KCFVRVESEKG	Disrupts interaction between FOXP3 and NFAT1	Hepatocellular carcinoma, Lung cancer	^80^
SAH-BCL9B	β-catenin	LSQEQLEHRERSLXTLRXIQRBLF ※ Amino acids at position X are stapled	Blocking binding of β-catenin/B-cell lymphoma 9	Colorectal adenocarcinoma, Multiple myeloma	^81^
hsBCL9CT-24	β-catenin	Ac-LQTLRXIQRXL-2-Nal ※ Amino acids at position X are stapled	Blocking binding of β-catenin/B-cell lymphoma 9	Adenocarcinoma, Colon cancer, Lung cancer, Breast cancer	^83^
P144	TGF-β1	TSLDASIIWAMMQN	Inhibiting the TGF-β signaling pathway	T-cell lymphoma	^84^
P17	TGF-β1	KRIWFIPRSSWYERA	Inhibiting the TGF-β signaling pathway	T-cell lymphoma	^85^
Fc-TPP-11	NRP-1	HTPGNSKPTRTPRR	Antagonizing NRP-1	Colon cancer, Melanoma	^87^
Peptide R29	CXCR4	Ac-RA-cyclic[DCRFFC]	Antagonizing CXCR4	In vitro only	^90^

P60 is a peptide that binds to FOXP3 and prevents its nuclear translocation. Lozano et al. used phage display screening and synthesized P60 (RDFQSFRKMWPFFAM), a 15-amino acid sequence that binds well to FOXP3. P60 penetrates cell membranes, binds to FOXP3, prevents nuclear translocation, reduces FOXP3’s ability to inhibit transcription factors NF-κB and NFAT, and impairs Treg activity^76^. CM-1315 is a high-tech product of P60 with improved FOXP3-binding ability and metabolic stability via modification of several base sequences of P60 to form a ring^77^. As peptides can penetrate all cells, they require a high dose to exert an antitumor effect. Lozano et al. conjugated a CD28 aptamer^78^ to P60 to address this drawback. CD28 is a T-cell-specific receptor that is abundant in Tregs. In an in vivo mouse experiment, when CD28Apt-P60 was coadministered with the AH1 vaccine, an antitumor effect on CT26 was shown at a lower dose than with free P60^79^.

FOXP3 393–403 is derived from FOXP3 and responsible for DNA binding. FOXP3 393–403 disrupts interaction between FOXP3 and NFAT1, enhances expression of proinflammatory cytokines and downregulates immunosuppressive molecules^80^.

SAH-BCL9B is an inhibitory peptide that blocks binding of β-catenin/B-cell lymphoma 9 (BCL9). Mutations and overexpression of beta-catenin are associated with many cancers. Kawamoto et al. analyzed the crystal structure of the BCL9 complex and developed this BCL9-derived peptide^81^. Tumor-specific Wnt/β-catenin signaling promotes immune evasion of tumor cells, enhances Treg production, inhibits CD8 + T-cell activation and invasion, converts DCs to an immunoregulatory phenotype, and differentiates CD8 + T cells into effector cells^82^. When SAH-BCL9B was administered in vivo, the Treg population in tumors decreased, whereas the populations of CD8+ effector T cells and CD103+ DCs increased^81^. Feng et al. synthesized hsBCL9CT-24 to improve the activity of SAH-BCL9B based on the BCL9 homology domain 2. Because hsBCL9CT-24 has a stronger binding affinity for β-catenin than BCL9-HD2A, it effectively reduces Tregs and increases DCs, promotes cytotoxic T-cell infiltration into a tumor, and shows a strong synergistic effect with anti-PD-1^83^.

P144 and P17 are peptide inhibitors that act on the TGF-β signaling pathway. P144 (TSLDASIIWAMMQN) is hydrophobic and forms the TGF-β1 binding region of human type III TGF-β1 receptor (TGFβRIII)^84^. P17 (KRIWFIPRSSWYERA) is a TGF-β-binding peptide identified by phage display screening^85^. TGF-β is produced at high levels by Tregs and suppresses the immune response by downregulating activity of T cells and natural killer cells. Llopiz et al. found that administration of P144 and P17 in combination with administration of the adjuvant molecule poly (I: C) and an agonist anti-CD40 antibody to mice bearing T-cell lymphoma increases antitumor immune responses^86^.

Fc-TPP-11 is synthesized by fusion of the NRP-1 binding peptide and the immunoglobulin Fc region of an antiangiogenic agent^87^. NRP1 is a membrane-bound coreceptor to a tyrosine kinase receptor for vascular endothelial growth factor and semaphorin family member^88^. Jung et al. demonstrated that Fc-TPP-11 inhibits Treg proliferation and function by antagonizing NRP-1. Fc-TPP-11 selectively suppresses intratumoral Treg function without compromising peripheral Treg function^89^.

Peptide-R29 is a novel CXCR4 antagonist derived from peptide R with higher avidity and stability. Santagata et al.^90^ demonstrated that CXCR4 is highly expressed in Tregs and that CXCR4 antagonism induced by a novel peptide antagonist, peptide-R29, efficiently reverses Treg suppression of effector T-cell proliferation.

## Small interfering RNA

siRNA is a double-stranded RNA (dsRNA) molecule 21–23 nucleotides in length that specifically causes RNA interference (RNAi), a posttranscriptional method of silencing gene expression. siRNA-based therapies have been developed for 20 years, and to date, four siRNA formulations have been FDA-approved for management of rare metabolic diseases, namely, patisiran, givosiran, lumasiran, and inclisiran^91^.

siRNAs exert their effects at the posttranscriptional level. The dsRNA is unwound into a single-stranded siRNA (guide strand) by an RNase III-like enzyme called Dicer, and the siRNA guide strand is loaded into a multiprotein component complex called the RNA-induced silencing complex (RISC). Once the RISC complex and the target mRNA are aligned, the mRNA is cleaved through the action of the catalytic RISC protein, a member of the Argonaute family (Ago2)^92^.

siRNA development has promoted new therapeutic approaches for a variety of diseases, as it has enabled selective inhibition of expression of specific genes and downregulation of specific proteins in the body through the design of custom oligonucleotide molecules using target mRNA transcript sequences. However, clinical application of siRNA-based therapeutics has been limited because of their off-target effects of rapid degradation and renal clearance, low cellular release, and evasion of intracellular uptake. In addition, negatively charged RNAi molecules have poor cellular uptake because cell membranes are composed of negatively charged bilayers of phospholipids and functional proteins^93^.

Therefore, an efficient delivery ‘carrier’ that can prevent siRNA degradation in vivo and deliver siRNA specifically to the cytoplasm of target cells is needed. Considering the characteristics of siRNAs, effective delivery systems for target cells have been investigated. Viruses, cationic lipids, polymers, and nanoparticles have been proposed as various approaches for in vivo siRNA delivery (Fig. 2)^94^.

## siRNA conjugates

siRNA conjugates are one of the most promising approaches involving covalent association of siRNAs with biomolecules (lipophilic molecules, antibodies, aptamers, ligands, peptides, or polymers). GalNAc siRNA conjugates have been approved by the FDA for treatment of acute hepatic porphyria^95,96^. siRNA conjugates are classified as aptamer-siRNA, peptide-siRNA, carbohydrate-siRNA, nanostructured material-siRNA conjugates, lipid-siRNA and polymer-siRNA^96^.

Aptamers are single-stranded oligonucleotides that recognize their targets through their unique three-dimensional complementarity. Because they are similar to mAbs due to their high affinity and specificity for the target molecule, they are called nucleic acid antibodies. However, they have several advantages over mAbs, including little to no immunogenicity or toxicity, a longer shelf life, lower production costs, and lower batch-to-batch variability. They have been utilized for the targeted delivery of siRNA to various cells (Fig. 2)^97,98^.

Peptides are suitable for siRNA delivery owing to their easy-to-define structures and various biological functions. High-yield peptide-siRNAs can be produced via various linkages, such as disulfide bonds, amide bonds, thioether bonds, thiol-maleimide bonds, the carboxy terminus of peptides, or additional cysteine residues. Peptide-siRNA conjugates can include CPP, CTP, soluble peptides, and carbohydrate peptides^96^.

Nanostructures are engineered structures with features at a nanoscale of fewer than 100 nanometers and include nanotextured surfaces, nanoparticles, nanotubes, and more complex nanoscale structures. They are readily taken up by target cells, act as specific ligands for receptors explicitly overexposed on the cell surface, avoid endosomes, and are nontoxic to healthy cells. Therefore, they can be useful carriers for siRNA conjugates for cancer treatment^99^. Nanostructure materials include gold nanoparticles, carbon nanotubes, magnetic nanoparticles, quantum dots, and mesoporous silica nanoparticles (Fig. 2)^100^.

## siRNA conjugates for targeting Tregs in the TME

CTLA4apt-STAT3 siRNA is a siRNA conjugate in which CTLA4 binding RNA aptamer and mouse STAT3 siRNA are linked. FOXP3+ Tregs in the TME are a significant cause of tumor-induced immunosuppression and highly express CTLA4^101^, and the transcription factor STAT3 is necessary and sufficient for Treg development. Inhibition of STAT3 signaling has been shown to inhibit tumor growth and improve survival; therefore, it is a molecular target for cancer treatment^102^. Herrmann et al. synthesized CTLA4apt-STAT3 siRNA for gene silencing and demonstrated blocking of tumor Treg accumulation and inhibition of tumor growth in various mouse tumor models^17^.

NP_siCTLA-4_ is a nanostructure material-siRNA conjugate in which siRNA-targeting CTLA-4 mRNA is surrounded by nanoparticles composed of PEG5k–PLA11k and BHEM-Chol^103^. Li et al. constructed NP_siCTLA-4_ to promote T-cell activation and proliferation. NP_siCTLA-4_ delivers CTLA-4-siRNA to CD4+ and CD8 + T-cell subsets in the TME while reducing the ratio of Tregs among TILs. In addition, NP_siCTLA-4_ effectively inhibits tumor growth and extends survival time in mouse models of melanoma^18^.

Hybrid SNPs are spherical nucleotide nanoparticles (SNPs) loaded with a CTLA-4-siRNA aptamer (cSNP) and PD-1 siRNA (pSNP) in a nanoparticle comprising an amphiphilic polymer of PLGA-S-S-PEG as the core and the cationic lipid DOTAP. Zhang et al. designed a hybrid SNP that combined the blocking strategies of CTLA-4 and PD-1. Hybrid SNPs show synergistic immunostimulatory effects by blocking CTLA-4 and PD-1, partially regulating the immunosuppressive function of Tregs and allowing effector T-cell expansion. Antitumor efficacy was demonstrated by inhibiting the growth of melanoma tumors and colorectal adenocarcinomas in mice^19^.

Manrique-Rincón et al. created an aptamer chimera by linking an aptamer that binds to 4-1BB, which is abundant in the Treg cell membrane, to a small antisense RNA (sasRNA) targeting FOXP3. The authors exploited a transcriptional gene silencing mechanism to suppress *Foxp*3 expression using sasRNA. Unlike posttranscriptional gene silencing, which represses mRNA expression, transcriptional gene silencing is driven by promoter or enhancer regions and is associated with DNA methylation. They showed that aptamer-sasRNA-mediated transcriptional gene silencing of Foxp3 promotes an antitumor response in combination treatment using a melanoma-bearing mouse model^28^.

## Conclusion

Existing drug conjugates have been developed to directly remove cancer cells. However, as current cancer treatments target immune-suppressive cells in the tumor microenvironment, drug conjugates are developing accordingly. Although the function of each moiety is known, when they are combined, the result may cause a synergistic effect beyond expectation or may have unexpected side effects.

Due to advanced synthesis technology, delivery efficiency and drug efficacy have been improved and can lower toxicity and side effects. Diverse conjugates with new drug-targeting molecules are increasing, and clinical trials with drug conjugates targeting immune-suppressive cells appropriately are driving innovative therapeutic design. This approach would be a promising strategy for overcoming the limitations of current anticancer drugs and specifically targeting the immunosuppressive TME.
